# Soluble Fiber Dextrin and Soluble Corn Fiber Supplementation Modify Indices of Health in Cecum and Colon of Sprague-Dawley Rats

**DOI:** 10.3390/nu5020396

**Published:** 2013-02-04

**Authors:** Brenda K. Knapp, Laura L. Bauer, Kelly S. Swanson, Kelly A. Tappenden, George C. Fahey, Maria R. C. de Godoy

**Affiliations:** 1 Department of Animal Sciences, University of Illinois, 1207 W. Gregory Drive, Urbana, IL 61801, USA; E-Mails: bknapp11@gmail.com (B.K.K.); llbauer@illinois.edu (L.L.B.); ksswanso@illinois.edu (K.S.S.); gcfahey@illinois.edu (G.C.F.); 2 Department of Food Science and Human Nutrition, University of Illinois, 905 S. Goodwin Avenue, Urbana, IL 61801, USA; E-Mail: tappende@illinois.edu

**Keywords:** cecal fermentation, histomorphology, soluble fiber dextrin, soluble corn fiber

## Abstract

The objective of this study was to evaluate health outcomes resulting from dietary supplementation of novel, low-digestible carbohydrates in the cecum and colon of Sprague-Dawley rats randomly assigned to one of four treatment groups for 21 days: 5% cellulose (Control), Pectin, soluble fiber dextrin (SFD), or soluble corn fiber (SCF). Rats fed Pectin had a higher average daily food intake, but no differences in final body weights or rates of weight gain among treatments were observed. No differences were observed in total short-chain fatty acid (SCFA) or branched-chain fatty acid (BCFA) concentrations in the cecum and colon of rats fed either SFD or SCF. The SFD and SCF treatments increased cecal propionate and decreased butyrate concentrations compared to Control or Pectin. Pectin resulted in increased BCFA in the cecum and colon. Supplementation of SFD and SCF had no effect on cecal microbial populations compared to Control. Consumption of SFD and SCF increased total and empty cecal weight but not colon weight. Gut histomorphology was positively affected by SFD and SCF. Increased crypt depth, goblet cell numbers, and acidic mucin were observed in both the cecum and colon of rats supplemented with SFD, SCF, and Pectin. These novel, low-digestible carbohydrates appear to be beneficial in modulating indices of hindgut morphology when supplemented in the diet of the rat.

## 1. Introduction

Dietary fiber as a promoter of healthy gut function and other health benefits is well recognized [1]. However, most of the population of the United States consumes less than half the recommended concentration of dietary fiber daily [2]. This has led to a demand for the development of novel carbohydrates that have functional properties similar to those of dietary fiber but that may be incorporated more easily into a wider array of solid and liquid food matrices. 

One class of carbohydrates, low-digestible carbohydrates, is becoming popular as a food ingredient, not only due to their potential to improve both the physical and chemical properties of foods, but also due to possible health benefits associated with their consumption that are similar in nature to those of dietary fiber [3]. Low-digestible carbohydrates are low molecular weight carbohydrates that resist hydrolytic activity of human digestive enzymes [4,5,6]. They pass into the colon where they are substrates for complete or partial fermentation by colonic microbiota. Fermentation results in short-chain fatty acids (SCFA) that provide colonic cells with energy and lower pH of luminal contents, stimulating a healthy environment for beneficial bacteria. Low-digestible carbohydrates also may beneficially impact the morphology of the gastrointestinal tract, especially through modulation of the mucosal layer. This layer is primarily composed of mucin glycoproteins synthesized and secreted by goblet cells that serve as a protective barrier for the epithelial cells [7]. Modulation of the mucosal layer may positively or detrimentally affect this barrier and, thus, the health of the gastrointestinal tract. 

Two novel, low-digestible carbohydrates are soluble fiber dextrin (SFD) and soluble corn fiber (SCF). Soluble fiber dextrin is an indigestible dextrin produced when corn starch is treated with heat and acid, and SCF is produced by isolating an oligosaccharide-rich fraction from corn syrup. Both of these novel, low-digestible carbohydrates are produced in such a way that branching and the number of α-1,6-glycosidic bonds are increased [8,9]. Soluble fiber dextrin and SCF have been reported to have a decreased *in vitro* hydrolytic digestion. Also, they attenuate glycemic and insulinemic responses and have reduced energy values [10]. However, little research exists regarding these novel, low-digestible carbohydrates on indices of gut health.

The objective of this study was to determine the effects of supplementation of SFD and SCF on select indices of gut health. This was determined by measuring pH, SCFA concentrations, and microbial populations in the cecum and/or colon of rats. Total and empty cecal and colonic mass and crypt and goblet cell measurements also were taken to determine the impact of these low-digestible carbohydrates on gut morphology. It was hypothesized that SFD and SCF would enhance fermentative processes in the hindgut, positively affecting intestinal microbiota and exerting trophic effects on gut morphology.

## 2. Experimental Section

### 2.1. Animals

Forty male Sprague-Dawley rats (average initial weight, 174 ± 11 g; 6 weeks of age) were purchased from Harlan Laboratories, Inc. (Indianapolis, IN, USA). Rats were housed individually in stainless steel wire-bottom cages in a temperature and humidity controlled facility with 12 h light and dark cycles. Prior to the experiment, rats were fed for 7 days on an AIN-93G diet [11]. Rats were given free access to water. All animal care procedures were approved by the University of Illinois Institutional Animal Care and Use Committee before initiation of the experiment.

### 2.2. Experimental Design and Treatments

Rats were randomly assigned to one of four dietary treatments (10 rats/treatment) after the adaptation period of 7 days. Rats were given free access to pelleted diets. Four dietary treatments were utilized in this study: a control diet that was the AIN-93G diet with 5% cellulose (Control), a positive control that consisted of the AIN-93G diet with 5% pectin (high-methoxy pectin, TIC Gums, White Marsh, MD, USA) substituted for cellulose (Pectin), a treatment that consisted of the AIN-93G diet with 5% soluble fiber dextrin (Nutriose, Roquette, Keokuk, IA, USA) substituted for cellulose (SFD), and a treatment that consisted of the AIN-93G diet with 5% soluble corn fiber (Promitor^®^, Tate & Lyle, Decatur, IL, USA) substituted for cellulose (SCF). All diets were prepared by Research Diets, Inc. (New Brunswick, NJ, USA). The ingredient and chemical composition of the diets is listed in Table 1. The duration of the study was 21 days. Food intake was determined daily and body weights were measured weekly.

**Table 1:** Ingredient and chemical composition of diets containing select dietary fibers and fed to rats.

Item	Control	Pectin	SFD ^1^	SCF ^2^
*Ingredient composition*	% of diet			
Cornstarch	39.75	39.75	39.75	39.75
Casein	20.00	20.00	20.00	20.00
Maltodextrin	13.20	13.20	13.20	13.20
Sucrose	10.00	10.00	10.00	10.00
Soybean oil	7.00	7.00	7.00	7.00
Cellulose	5.00	0.00	0.00	0.00
Pectin	0.00	5.00	0.00	0.00
SFD	0.00	0.00	5.00	0.00
SCF	0.00	0.00	0.00	5.00
Mineral mix ^3^	3.50	3.50	3.50	3.50
Vitamin mix ^4^	1.00	1.00	1.00	1.00
L-Cystine	0.30	0.30	0.30	0.30
Choline bitartrate	0.25	0.25	0.25	0.25
Dye	0.005	0.005	0.005	0.005
*t*-Butylhydroquinone	0.0014	0.0014	0.0014	0.0014
*Chemical composition*	% of diet			
Dry matter (DM)	90.6	90.4	89.1	89.6
	% DM basis			
Organic matter	97.4	97.3	97.4	97.4
Crude protein	18.7	19.5	19.3	19.2
Total dietary fiber	5.9	5.3	2.0	2.2
Acid hydrolyzed fat	6.9	6.9	7.0	7.0
Gross energy, kcal/g	4.7	4.7	4.7	4.7

^1^ Soluble fiber dextrin; ^2^ Soluble corn fiber; ^3^ Mineral mix = AIN-93G-MX. Mineral (g/kg): Calcium carbonate, 357.00; Potassium phosphate, 196.00; Potassium citrate, 70.78; Sodium chloride, 74.00; Potassium sulfate, 46.60; Magnesium oxide, 24.00; Ferric citrate, 6.06; Zinc carbonate, 1.65; Sodium meta-silicate, 1.45; Manganous carbonate, 0.63; Cupric carbonate, 0.30; Chromium potassium sulfate, 0.28; Boric acid, 0.08; Sodium fluoride, 0.06; Nickel carbonate, 0.03; Lithium chloride, 0.02; Sodium selenate, 0.01; Potassium iodate, 0.01; Ammonium paramolybdate, 0.008; Ammonium vanadate, 0.007; Powdered sucrose, 221.03; ^4^ Vitamin mix = AIN-93G-VX. Vitamin (mg/kg) (except as noted): Nicotinic acid, 3.00; Ca pantothenate, 1.60; Pyridoxine, 0.70; Thiamin, 0.60; Riboflavin, 0.60; Folic acid, 0.20; Biotin, 0.02; Vitamin B_12_, 2.50; Vitamin E (500 IU/g), 15.00; Vitamin A (500,000 IU/g), 0.80; Vitamin D_3_ (400,000 IU/g), 0.25; Vitamin K, 0.08; Powdered sucrose, 974.65.

### 2.3. Sample Collection

On day 21, rats were euthanized by placement in a CO_2_ chamber. A ventral midline incision then was made and the cecum and colon were removed. Immediately after removal, cecum and colon with contents were weighed to determine total weight. pH of cecal and colonic contents was taken using a Beckman pH meter and electrode (Beckman Instruments, Inc., Fullerton, CA, USA). Aliquots of cecal and colon contents then were taken for DM, SCFA, and microbiota analysis. The SCFA aliquots were acidified with 5 mL 2 N HCl before storing at −20 °C. The aliquot for microbial analysis was sealed in a sterile cryovial, snap frozen in liquid nitrogen, and stored at −80 °C. No colonic contents were collected for microbiota analysis due to insufficient amounts of colonic digesta. 

Following removal of the appropriate samples, the tissues were cleaned with water, blotted dry, and weighed to determine empty cecum and colon weights. Total cecal and colonic contents were calculated as total tissue weight with contents minus empty tissue weight. Cecal and colonic tissue from rats was collected and fixed in phosphate buffered formalin for histomorphological analysis.

### 2.4. Chemical Analysis

Diet samples were analyzed for dry matter (DM), organic matter (OM) [12], Leco N [12], acid hydrolyzed fat (AHF) [13,14], and gross energy (GE) (Parr Instrument Co., Moline, IL, USA). Diet samples also were analyzed for total dietary fiber (TDF) content [15]. All procedures were performed in duplicate. To maintain quality control during chemical analysis, the error between duplicate samples was determined and, if it exceeded 5%, the assay was repeated. Fresh cecal and colonic contents were analyzed for DM and pH (as indicated above), and SCFA using gas chromatography [16]. Briefly, acetate, propionate, butyrate, isobutyrate, isovalerate, and valerate concentrations were determined on the supernatant of acidified cecal and colonic contents using a Hewlett-Packard 5890A Series II gas chromatograph (Palo Alto, CA, USA) and a glass column packed with 10% SP-1200/1% H_3_PO_4_ on 80/100+ mesh Chromosorb WAW (Supelco, Bellefonte, PA, USA).

### 2.5. Microbial Analysis

Microbial populations were analyzed using methods described by Middelbos *et al.* [17] with minor modifications. Cecal digesta DNA was extracted from freshly collected samples that had been stored at −80 °C until analysis, using the repeated bead beater method described by Yu and Morrison [18] followed by a QIAamp DNA stool mini kit (Qiagen, Valencia, CA, USA) according to manufacturer’s instructions. Extracted DNA was quantified using a NanoDrop ND-1000 spectrophotometer (Nano-Drop Technologies, Wilmington, DE, USA). *Escherichia coli*, the *Bifidobacterium* genus, and the *Lactobacillus* genus were quantified using quantitative polymerase chain reaction (qPCR) and specific primers. Amplification was performed for each bacterial group within each sample according to the procedures of Deplancke and co-workers [19]. For amplification, 10 μL final volume containing 5 μL of 2× SYBR Green PCR Master Mix (Applied Biosystems, Foster City, CA, USA), 15 pmol of the forward and reverse primers of the bacteria of interest, and 5 ng of extracted cecal DNA were used. Pure cultures of each bacterium were used to create serial dilutions in triplicate of the targeted bacterial genus to obtain standard curves. Bacterial DNA was extracted from each dilution and amplified along with cecal DNA samples using a Taqman ABI PRISM 7900HT Sequence Detection System (Applied BioSystems, Foster City, CA, USA). Colony forming units (cfu) of each standard curve serial dilution were determined previously by plating on specific agars. *E. coli* was grown on Luria-Bertani medium, *Lactobacillus* on Difco Lactobacilli MRS broth (Becton, Dickenson, and Co., Sparks, MD, USA), and *Bifidobacterium* on Difco Reinforced Clostridial Medium (Becton, Dickenson, and Co., Sparks, MD, USA). Cycle threshold values were plotted against the standard curves for quantification (cfu/g cecal contents) of the targeted bacterial DNA from cecal samples. 

### 2.6. Cecal and Colonic Histomorphology

Cecal and colonic sections from each rat were embedded in a paraffin block, sliced into 5 µm thick sections using a microtome, and stained. One set of slides was stained with alcian blue (AB) and periodic acid Shiff and counterstained with hematoxylin for determining crypt depth, goblet cell numbers, and mucin (acidic and neutral) components. Another set of slides was stained with high iron diamine (HID) and AB to determine sulfated and sialylated mucins; subtypes of acidic mucins. Slides were prepared and stained at the Department of Veterinary Biosciences Histology Laboratory, University of Illinois. Crypt depth, goblet cell counts, and mucin composition measurements were attempted on a minimum of 15 crypts per section. Data are presented as the average number of stained goblet cells per crypt. Digital images of tissues and measurements were taken using Axiovision LE software and an AxioCam MRc5 (Zeiss, Oberkochen, Germany).

### 2.7. Statistical Analyses

Data were analyzed as a completely randomized design using the Mixed Models procedure of SAS (SAS Institute, Inc., Cary, NC, USA). The model contained the fixed effect of diet and the random effect of rat. Differences among treatments were determined using a Fisher-protected least significant difference test with a Tukey adjustment to control for experiment-wise error. Reported pooled standard errors of the mean (SEM) were determined according to the Mixed Models procedure of SAS. Significant differences were accepted at a probability of *P* < 0.05.

## 3. Results

### 3.1. Diet Composition, Body Weight, and Food Intake

Dietary treatments were similar in DM, OM, CP, AHF, and GE composition (Table 1). Total dietary fiber concentrations were lower for the SFD and SCF diets because the TDF method used does not fully quantify low-molecular weight dietary fibers like SFD and SCF. The complete carbohydrate composition of SFD and SCF is presented in Knapp *et al.* [10]. 

Daily food intake, final body weights, and rate of weight gain are presented in Table 2. Initial body weights of the rats were similar among the groups (avg. 178.4 g) and, after 21 days on the experimental diets, the final body weights and rate of weight gain did not differ significantly. Daily food intake was approximately 16.5 g/day, with rats fed the Pectin diet having a higher (*P* < 0.05) daily food intake. 

**Table 2:** Daily food intake, rate of weight gain, and final body weights of rats fed select dietary fibers.

Item	Treatment				
	Control	Pectin	SFD ^1^	SCF ^2^	SEM ^3^
Daily food intake, g/day	16.5 ^a^	17.4 ^b^	16.1 ^a^	15.9 ^a^	0.26
Initial body weight, g	178.2	178.8	179.3	177.4	2.09
Final body weight, g	314.6	323.8	327.4	318.5	6.01
Rate of weight gain, g/day	4.6	4.9	5.0	4.5	0.22

^1^ Soluble fiber dextrin; ^2^ Soluble corn fiber; ^3^ Pooled SEM; ^a,b^ Means in the same row with different superscript letters are different (*P* < 0.05).

### 3.2. Cecal and Colonic Weight, pH, and Dry Matter Content

Total weight, empty weight, and pH values for cecum and colon are presented in Table 3. Total weight of the cecum was dramatically increased (*P* < 0.05) as a result of consumption of SFD and SCF. However, this effect was not noted in the colon where all treatments resulted in a similar total colon weight. Empty cecal weight was increased (*P* < 0.05) compared with the Control as a result of Pectin, SFD, and SCF consumption, with values for the latter two fibers being higher than that for Pectin. Empty colonic weight was unaffected by diet. Cecal and colonic pH values were lowered (*P* < 0.05) by SFD and SCF. Dry matter of cecal contents was greatest for Control and SCF, intermediate for SFD, and smallest for Pectin (*P* < 0.05). Colon content (% DM) did not differ among dietary treatments and it was quite variable as depicted by the large SEM.

**Table 3:** Cecal and colonic total and empty weights, pH, and dry matter content values in rats fed select dietary fibers.

Item	Treatment				
	Control	Pectin	SFD ^1^	SCF ^2^	SEM ^3^
Total weight, g					
Cecum	3.1 ^a^	4.0 ^a^	6.7 ^b^	6.2 ^b^	0.38
Colon	1.8	1.5	1.3	1.5	0.16
Empty weight, g					
Cecum	0.9 ^a^	1.2 ^b^	1.6 ^c^	1.3 ^c^	0.08
Colon	0.9	0.9	0.9	0.9	0.07
Cecal pH	6.9 ^b^	6.7 ^b^	5.9 ^a^	6.0 ^a^	0.10
Colon pH	6.9 ^b^	6.9 ^b^	6.2 ^a^	6.1 ^a^	0.14
Cecal content, % DM	21.8 ^c^	15.0 ^a^	18.5 ^b^	22.1 ^c^	1.05
Colon content, % DM	31.3	28.4	28.4	33.9	6.77

^1^ Soluble fiber dextrin; ^2^ Soluble corn fiber; ^3^ Pooled SEM; ^a,b,c^ Means in the same row with different superscript letters are different (*P* < 0.05).

### 3.3. Histomorphology

Histomorphology data collected on rat cecum and colon are presented in Table 4. 

**Table 4:** Effect of select dietary fibers on cecum and colon histomorphology of rats.

Item	Treatment				
	Control	Pectin	SFD ^1^	SCF ^2^	SEM ^3^
**Cecum**					
Crypt depth, μm	164.6 ^a^	210.6 ^b^	208.8 ^b^	201.5 ^b^	5.46
**Goblet cells (*n*) per crypt**					
Total	12.6 ^a^	20.1 ^b^	19.8 ^b^	19.3 ^b^	1.48
Acidic mucin	7.1 ^a^	14.2 ^b^	13.9 ^b^	12.5 ^b^	1.22
Mixed (acidic/neutral)	5.7	7.5	6.9	6.9	0.41
**Mucin (*n*) per crypt**					
Sulfomucins	6.8	8.2	7.8	8.1	0.68
Sialomucins	5.9	6.2	6.7	6.0	0.44
**Colon**					
Crypt depth, µm	216.5 ^a^	257.5 ^b^	245.4 ^b^	242.7 ^b^	7.94
**Goblet cells (*n*) per crypt**					
Total	15.8 ^a^	25.0 ^b^	23.9 ^b^	24.3 ^b^	1.36
Acidic mucin	11.5 ^a^	21.4 ^b^	19.4 ^b^	20.1 ^b^	1.28
Mixed (acidic/neutral)	3.8	4.5	5.2	4.9	0.32
**Mucin (*n*) per crypt**					
Sulfomucins	6.6 ^a^	13.3 ^b^	10.5 ^b^	10.9 ^b^	1.00
Sialomucins	10.7	10.2	9.9	10.2	0.92

^1^ Soluble fiber dextrin; ^2^ Soluble corn fiber; ^3^ Pooled SEM; ^a,b^ Means in the same row with different superscript letters are different (*P* < 0.05); (*n*) = the average number of stained goblet cells per crypt.

Crypt depth in both cecum and colon was increased (*P* < 0.05) in rats fed the Pectin, SFD, and SCF compared with Control. A similar pattern for goblet cell number was noted. In both cecum and colon, supplementation of Pectin, SFD, and SCF increased (*P* < 0.05) goblet cell numbers compared to Control. 

The majority of goblet cells found in both cecum and colon crypts were found to be comprised of acidic mucin, and increased (*P* < 0.05) acidic mucins were found in rats fed Pectin, SFD, and SCF. These were found concentrated towards the bottom of the crypts. No goblet cells composed of only neutral mucin were observed in cecal or colonic crypts for any treatment; however, goblet cells comprised of a mixture of both acidic and neutral mucins were observed. These cells stained purple, indicating that both types of mucins were present [20]. No differences between cecum and colon or among dietary treatments were observed for mixed goblet cells. Fermentable substrates resulted in more sulfomucins relative to Control in colon but not in cecum.

### 3.4. Microbial Concentrations

No differences among treatments were noted in cecal concentrations of *Bifidobacterium* spp., *Lactobacillus* spp., or *Escherichia coli* (data not shown). Average concentration values across treatments for these microbiota were 9.7, 11.4, and 11.7 log10 cfu/g cecal DM, respectively. 

### 3.5. Short-Chain Fatty Acids and Branched-Chain Fatty Acids

Fermentative end-product concentrations in cecal and colonic contents are presented in Table 5. Pectin resulted in increased (*P* < 0.05) acetate concentrations in cecal contents compared to the other treatments. Propionate concentrations were highest (*P* < 0.05) for SFD, with SCF and Pectin having lower (*P* < 0.05) concentrations. Cecal concentrations of butyrate were lowest (*P* < 0.05) for the SFD and SCF treatments. Pectin supplementation resulted in the highest (*P* < 0.05) total cecal SCFA concentration among treatments. Supplementation of SFD and SCF resulted in similar total SCFA when compared to the Control diet. 

Cecal isobutyrate, valerate, and total BCFA concentrations were lower (*P* < 0.05) with supplementation of both SFD and SCF compared to either Control or Pectin treatments. Isovalerate concentrations were lower (*P* < 0.05) for the SFD and SCF treatments compared to Pectin. Pectin resulted in the highest (*P* < 0.05) cecal concentrations of total BCFA for all dietary treatments. 

Colonic SCFA concentrations were lower overall compared to those in cecal contents. Acetate and total SCFA concentrations were higher (*P* < 0.05) for the Pectin treatment compared to the SCF treatment. Soluble fiber dextrin resulted in higher (*P* < 0.05) propionate concentrations compared to Control. Similarly to cecal SCFA, butyrate concentrations were higher (*P* < 0.05) for the Pectin and Control treatments compared to SFD and SCF treatments.Concentrations of BCFA were lower in the colon compared to concentrations in the cecum. Pectin resulted in higher (*P* < 0.05) concentrations of isobutyrate than did SFD. Isovalerate concentrations were higher (*P* < 0.05) for Pectin compared to Control and SCF. Pectin resulted in the highest (*P* < 0.05) concentrations of valerate and total BCFA compared with the other treatments. 

**Table 5:** Concentrations of short-chain fatty acids (SCFA) and branched-chain fatty acids (BCFA) in cecal and colonic contents of rats fed select dietary fibers.

Item	Treatment				
	Control	Pectin	SFD ^1^	SCF ^2^	SEM ^3^
**Cecal SCFA, µmol/g ^4^**					
Acetate	192.3 ^a^	460.9 ^b^	206.8 ^a^	171.6 ^a^	20.46
Propionate	53.0 ^a^	88.1 ^b^	113.5 ^c^	90.4 ^b^	6.33
Butyrate	52.8 ^b^	60.2 ^b^	13.6 ^a^	14.0 ^a^	5.15
Total SCFA	298.2 ^a^	609.2 ^b^	333.9 ^a^	276.0 ^a^	26.17
**Cecal BCFA, µmol/g ^4^**					
Isobutyrate	4.5 ^b^	5.3 ^b^	2.7 ^a^	2.2 ^a^	0.44
Isovalerate	4.6 ^a,b^	5.6 ^b^	4.0 ^a^	3.5 ^a^	0.35
Valerate	5.0 ^b^	8.1 ^c^	1.5 ^a^	1.9 ^a^	0.41
Total BCFA	14.1 ^b^	19.0 ^c^	8.2 ^a^	7.6 ^a^	1.04
**Colonic SCFA, µmol/g ^4^**					
Acetate	118.1 ^a,b^	214.9 ^b^	100.7 ^a,b^	81.7 ^a^	29.94
Propionate	29.7 ^a^	50.8 ^a,b^	73.9 ^b^	34.1 ^a,b^	11.09
Butyrate	30.3 ^b^	43.6 ^b^	6.7 ^a^	6.3 ^a^	5.86
Total SCFA	178.8 ^a,b^	309.1 ^b^	180.9 ^a,b^	121.7 ^a^	42.70
**Colonic BCFA, µmol/g ^4^**					
Isobutyrate	2.3 ^a,b^	3.3 ^b^	1.2 ^a^	1.6 ^a,b^	0.45
Isovalerate	2.3 ^a^	4.5 ^b^	3.0 ^a,b^	2.2 ^a^	0.54
Valerate	2.4 ^a^	4.9 ^b^	0.9 ^a^	1.3 ^a^	0.55
Total BCFA	6.9 ^a^	12.8 ^b^	5.0 ^a^	5.0 ^a^	1.43

^1^ Soluble fiber dextrin; ^2^ Soluble corn fiber; ^3^ Pooled SEM; ^4^ Values are expressed on a dry matter basis; ^a,b,c^ Means in the same row with different superscript letters are different (*P* < 0.05).

## 4. Discussion

Rats consuming the SCF diet developed diarrhea soon after starting the treatment, but did not significantly decrease food intake or lose weight. Consumption of Pectin and SFD also resulted in looser stools by the end of the study, but not to the extent experienced by rats fed SCF. The % DM of the cecal and colon contents is reflective of the last day of experiment, which agrees with the fecal consistency observation. Weaver *et al.* [21] supplemented SCF and SFD to rats at 10% of the diet and found that they also developed loose stools. The test carbohydrates then were reduced to 5% dietary concentration and loose stools persisted, as was the case in the current study. Low-digestible carbohydrates such as SCF and SFD can result in tolerance issues such as diarrhea when consumed for a period of time [22,23,24].

Weaver *et al.* [21] also found that supplementation with SCF, SFD, and other novel fibers increased cecum weight compared to cellulose. In that study, supplementation of 4% SCF and SFD resulted in a cecum weight of 5.58 g, similar to what was found in the current study (cecal weight of 6.15 g and 6.72 g, respectively). Other research has demonstrated that ingestion of low-digestible carbohydrates resulted in increased cecum weights of rats [25,26,27,28]. The increased cecal weight likely is due to increased epithelial cell proliferation from the trophic effects of SCFA [29]. The major differences in organ weights were noted only for cecum and not for colon. This is probably due to the fact that the major site of fermentation for rodents is the cecum and not the colon as in humans. The decreased cecal pH is due to increased SCFA production at that site. Even though an increase in SCFA production was not always followed by a decrease in pH, this could potentially be a result of the production of lactic acid (not measured in this study) that would lead to pH change, but would not be accounted for in the total SCFA production. 

Increased crypt depth as a result of dietary supplementation of low-digestible carbohydrates is a beneficial morphological effect. The crypts contain intestinal stem cells, the principal site of cell proliferation in the intestinal mucosa, and increased depth is associated with increased rate of turnover of intestinal mucosal cells [30,31]. Several studies have shown that pectin and other dietary fibers increase crypt depth throughout the intestinal tract [31,32,33]. However, pectin has been reported to simultaneously increase crypt depth and decrease villus height of the small intestine [32].

The increase in goblet cells per crypt may have a positive impact on gut health by increasing the thickness of the mucous layer of the large bowel. Other studies have reported increased goblet cell numbers in rats fed fermentable fibers including fructans and galactooligosaccharides [31,34,35,36,37]. Acidification of large intestinal contents is postulated to stimulate mucus synthesis and secretion [38] and could perhaps explain the increased numbers of goblet cells with the dietary treatments tested in this experiment. It has been suggested that acidic mucins protect against bacterial translocation because sulfated mucins (sulfomucins) in particular appear to be less degradable by bacterial glycosidases and host proteases [39]. Rats fed diets supplemented with low-digestible, inulin-type fructans have been shown to modulate mucins in the intestinal tract by increasing acidic mucins, especially the protective sulfomucins [31,36,40]. Alterations in the mucosal architecture and amounts of sulfomucins and sialomucins could have important effects on the gut mucosal barrier and health maintenance of the gut. Sulfomucins or sialomucins were found in both the cecal and colonic crypts. In the cecum, no differences among dietary treatments were observed. However, for the colonic crypts, diets supplemented with Pectin, SFD, and SCF had higher numbers of sulfomucins compared with Control. 

Soluble corn fiber has been shown to affect microbial concentrations *in vitro*. Maathuis *et al.* [41] reported a 2-fold increase in *Bifidobacterium* spp. using SCF in a validated dynamic computer-controlled *in vitro* model of the human proximal large intestine (TIM-2), where soluble corn fiber was fermented for 36 h, with a feeding rate of 10 g of test substrate per 24 h period. A bifidogenic response also was found in a human *in vivo* study where healthy men were supplemented with 21 g/day of SCF for 21 days [42]. This dose of SCF was found to increase (*P* < 0.05) fecal concentrations of *Bifidobacterium* spp. compared with the non-fiber control (from 6.9 log10 cfu/g to 8.2 log10 cfu/g), but did not have any effect on *Lactobacillus* spp. or *Escherichia coli* populations. Pasman *et al.* [43] found that neither 30 nor 45 g/day of SFD increased *Lactobacillus* spp. in feces compared with a maltodextrin control in a human study. Neither carbohydrate affected microbiota concentrations in rat cecum, indicating potential differences in responses due to experimental design such as *in vivo vs. in vitro* model, species, dose of test substrates, and fermentation period. 

The lack of difference between SFD and SCF as regards cecal SCFA compared to Control may be due to the increased cecal volume of rats consuming SFD and SCF, thus leading to a dilution effect for SCFA in cecal contents. Colonic SCFA concentrations were lower than those observed for cecal SCFA production. However, a similar pattern was observed, with Pectin treatment showing greater SCFA production. Small but similar amounts of colonic contents were found for all dietary treatments; thus, few differences among treatments were observed. The Control resulted in higher (*P* < 0.05) butyrate concentrations compared to SFD and SCF. The cecum is the main fermentative organ for the rat; therefore, a higher production of SCFA is to be expected at this site when compared to the colon. 

Neither of the novel, low-digestible carbohydrates were butyrogenic in contrast to Control and Pectin treatments. Weaver *et al.* [21] found a similar response to SCF and SFD in cecal SCFA concentrations in rats. The supplemental SCF and SFD did not increase butyrate concentrations compared to a cellulose control when supplemented at 4% of the diet. Stewart *et al.* [44] found that supplementation of 12 g/day SFD and SCF to human subjects resulted in no differences in fecal SCFA concentrations compared with a maltodextrin control. Soluble corn fiber has been supplemented at 21 g/day to human subjects and, similar to results with rats, fecal butyrate concentrations were not increased compared with a control [42]. 

In general, colonic BCFA concentrations for Control rats were similar to those for rats fed SFD and SCF. However, as regards cecal BCFA concentrations, Control tended to result in higher concentrations than did SFD and SCF. Overall, for both cecal and colonic BCFA, Pectin had higher values than did the other dietary treatments. Pectin increases the viscosity of digesta, which could decrease crude protein digestion, resulting in higher quantities of amino acids reaching the cecum and colon where they would be fermented, thus producing BCFA [45,46].

Although this research provides valuable information on the fermentative behavior and on the potential beneficial effects of SFD and SCF in gut health, a limitation of this study is that the cecum is the major fermentative site in the rat, whereas in humans it is the colon. Furthermore, in this study these substrates were incorporated into a semi-purified diet, which differs from how these products may be consumed by humans. Also, results observed from SFD and SCF are dependent on the brand of each fiber source used herein; therefore, outcomes might vary when using different sources of SFD and SCF. 

In summary, SFD and SCF both resulted in extensive fermentation in the cecum of rats. Dietary supplementation at the 5% level of the diet resulted in tolerance issues (loose stools) for the Pectin, SFD, and SCF treatments, but this did not affect food intake, body weight, or rate of weight gain. Diets containing SFD and SCF resulted in total cecal SCFA concentrations similar to those of Control diet. In general, Pectin resulted in higher concentrations of BCFA in cecal and colonic contents compared to SFD and SCF. Even though SFD and SCF did not result in increased butyrate concentrations, they nevertheless resulted in positive effects on cecal and colonic histomorphology. 

## 5. Conclusions

In conclusion, SFD and SCF have the potential to beneficially impact large bowel morphology. Both of these low-digestible carbohydrates increased cecal weight, increased cecal and colonic crypt depths, and had a positive effect on goblet cells and mucin composition. Even though SFD and SCF were fermented in the hindgut of rats, they do not appear to be butyrogenic or bifidogenic in the rat. Future research is warranted to determine the optimal dietary supplementation level of SFD and SCF to minimize tolerance issues and to still provide beneficial effects on gut histomorphology important for maintenance of the gut health. 
